# An optimal method for calculating an average screw axis for a joint, with improved sensitivity to noise and providing an analysis of the dispersion of the instantaneous axes

**DOI:** 10.1371/journal.pone.0275218

**Published:** 2022-10-17

**Authors:** Andrea Ancillao, Maxim Vochten, Arno Verduyn, Joris De Schutter, Erwin Aertbeliën

**Affiliations:** 1 Robotics Research Group, Department of Mechanical Engineering, KU Leuven, Leuven, Belgium; 2 Flanders Make, Core Lab ROB, KU Leuven, Leuven, Belgium; Universidad Nacional Autonoma de Nicaragua Leon, NICARAGUA

## Abstract

The instantaneous (ISA) and average (ASA) screw axes are techniques commonly adopted in motion analysis to functionally locate the rotation axis and center of rotation of a joint. Several approaches for calculating such axes were proposed in literature and the main limitations were identified as the need for using a threshold on angular displacements or velocities for handling the cases where the ISA is ill-defined and the need for a method for reliably estimating the center or rotation in limit cases, such as a purely rotational motion in the three-dimensional space. Furthermore, in many applications, such as in biomechanics, it is useful to statistically estimate the dispersion or variation of the ISA with respect to the ASA. In this paper we propose a novel method for estimating an ASA. Our method represents an improvement over previous methods as it: (i) exploits an optimization procedure based on the instantaneous differential kinematics (screw twist); (ii) removes the need for a threshold by introducing a weighting based on the norm of angular velocity; (iii) handles the singular cases where the position of the ASA is ill-defined by proposing a regularization term in the optimization. In addition, we proposed a method for estimating the uncertainty in the ASA calculation. The same quantities serve as a measure of the dispersion of the ISAs with respect to the ASA. The method was tested on real data and surrogate data: (i) a human gait analysis trial representing the motion of a knee, (ii) the experimental recording of the free swing motion of a mechanical hinge and (iii) synthetically generated motion data of a purely rotational (cylindrical) motion. The results showed that the new method had a lower sensitivity to noise, was able to reasonably handle the singular cases and provide a detailed analysis of ISA dispersion.

## Introduction

The calculation of the Instantaneous Screw Axis (ISA) is a common technique in motion analysis to analyze the motion of a joint. For example, in clinical motion analysis it is common to calculate the ISA of a hinge-like joint (e.g. the knee) during a controlled motion. In such a case, the motion and variations of the ISA were correlated to the functionality of the joint itself and to the healthiness of the ligaments [1–3]. The calculation of the ISA is based on the Mozzi–Chasles’ theorem, that allows to represent any rigid-body motion as a translation along an axis plus a rotation about the same axis [4, 5]. The literature refers to the ISA also as “helical axis”, “twist axis” or “axis of rotation” [6, 7].

The most common method to measure the motion of a joint is by using some motion capture technique (e.g. optoelectronic systems or inertial sensors) to track the motion of the segments composing the joint. The segments are usually assumed as rigid bodies [8]. Commonly the output of such measurements are the coordinates of skin-attached markers and/or the poses of the two segments expressed in the form of a homogeneous matrix [9]. Several techniques to compute the ISA, based on these data, were proposed in the literature. They can be divided into two main categories: (i) techniques based on the differences in pose between two configurations (angular displacements and/or variation of the homogeneous matrix between two time instants) and (ii) techniques based on the differential kinematics of the joint (angular velocity / screw twist). When the screw axis is computed in between any two given configurations it is also named “finite screw axis” [10]. Examples of the equations for calculating the screw axis based on poses/displacements are described in [10, 11]. Those are not discussed here as it goes beyond the scope of the present paper. Instead, when the calculation is based on the screw twist, the axis is often named “instantaneous” screw axis and the calculation is described in [7, 12, 13].

When analyzing the ISA over a biomechanical motion, it is often necessary to compare it to a reference to study the variations with respect to that reference.

In previous studies, a reference for the ISAs was obtained geometrically, for example using the condyle-to-condyle line [14, 15], the rotation axis of a prosthesis [16], or anatomical planes [17–19]. An alternative approach is to adopt a functional procedure to compute the reference axis, as several studies showed that a functional approach outperformed the geometrical one, especially for some ad-hoc applications, such as the design of prostheses [12, 20, 21]. Such an axis is conceptually the “mean” of the set of ISAs associated to a given motion. It is also addressed as “mean finite helical axis” [21] or “optimal flexion axis”. Several techniques for calculating the average axis were proposed in the literature. Most of them were based on optimization procedures [12, 22, 23] that aim to find: (i) the orientation (unit vector, ***n***) that is “the most parallel” to the set of ISAs and (ii) the point ***P*** closest to all the ISAs, i.e. the pseudo-intersection of the ISAs. The previously cited quantities may be calculated via a least-squares approach [12].

However, since the ISA is ill-defined, it should not be calculated when the norm of the angular velocity is below a threshold, often assumed as ~0.3 rad/s [12].

To avoid using a threshold on the angular velocity, [23] proposed a weighted approach to find the point that best approximates the pseudo-intersection of the ISAs, and the optimal orientation of an average axis, named “mean finite screw axis”, or MFSA. It was based on the finite screw axis between two poses observed at the *i-th* and *j-th* instants of the motion and it minimizes the root mean square distance between all the possible pairs of finite screw axes, with a weighting factor based on the sine of the angle between the considered axes. The approach of [21] is effective in that it produces a reference axis with low sensitivity to measurement noise without requiring the use of a threshold, but it is computationally slow since it requires the calculation of all the possible couples of instants in the set: *i*,*j*. In addition, the obtained reference axis is the mean of all possible finite screw axes, but not the average of the ISAs, which are a representation of the local differential kinematics. The latter is the focus of this paper. Another limitation of the previously cited methods is that the localization of the pivot point becomes unstable and less reliable for hinge-like motions. In the limit case of a perfect hinge, where ISAs are parallel to each other, the pivot point is undefined. Also, the ISA itself is ill-defined for low values of angular velocity [7]. Instead, the calculation of the pivot point becomes more accurate for joints having a spherical behavior [24, 25].

The calculation of an ASA is useful for many practical applications, such as: gait analysis, human motion analysis, robotics, motion representation, etc. and it may also serve as an approximation of the true rotation axis of joints that can be modelled as hinges [12]. From this follows the aim of the current work. Furthermore, from a biomechanical point of view, it is particularly important to quantify the dispersion or the deviation of the ISAs with respect to the ASA, that was correlated to the stability and healthiness of the joint [17, 25, 26].

## Aims

The aim of this work is to propose a novel optimal procedure for calculating the Average Screw Axis (ASA) of a joint motion based on the measured screw twist. The improvements with respect to the previous methods are: (i) implementation of an optimization procedure based on the instantaneous differential kinematics (screw twist); (ii) the use of weights corresponding to the magnitude of the angular velocity to handle cases where the ISA is ill-defined due to low velocities; (iii) the introduction of a regularization term in the optimal calculation of the ASA position to handle cases where the variation in ISA orientation is small and therefore the pseudo-intersection point of the set is ill-defined.

In addition, we propose a novel procedure for the analysis of the uncertainty occurring in the estimation of the ASA which also serves as a measure of the dispersion or variation of the ISAs with respect to the ASA.

Finally, we test our method and compare it against the current state-of-the-art methods. We exploit three datasets: (i) a human gait analysis trial representing the motion of a knee, (ii) the experimental recording of the free swing motion of a mechanical hinge and (iii) synthetically generated motion data of a purely rotational (cylindrical) motion.

## Materials and methods

This paper presents a method for calculating an optimal ASA for the motion of a joint based on the measured screw twist of the joint during the motion. Conceptually, the ASA represents the line that is the closest and the “most parallel” to a set of ISAs.

The screw twist (***tw***) characterizes the velocity of a rigid body with respect to a coordinate system (CS) as the rotational velocity vector **ω** and the translational velocity vector **v**_0_ of the point on the rigid body that instantaneously coincides with the origin of the reference CS:

$$tw=(\begin{array}{c} \omega \\ v_{0} \end{array}).$$ (1)

For a joint, the screw twist can then be used to represent the relative motion between the rigid segments composing the joint. The screw twist can be calculated based on *ΔT*, the difference between two consecutive relative poses *T* (homogeneous transformation matrices) between the segments composing the joint, according to the following equation [7, 27]:

$$\left(\begin{array}{cc} {\left[\omega \right]}_{x} & v_{0}\\ 0_{1x3} & 0 \end{array}\right)=\frac{1}{\Delta t}logm(\Delta T),$$ (2)

where *Δt* is the time step between consecutive samples and [***ω***]_x_ is the skew symmetric matrix formed out of the coordinates of ***ω*:**

$${\left[\omega \right]}_{x}=(\begin{array}{ccc} 0 & {-\omega }_{z} & \omega_{y}\\ \omega_{z} & 0 & {-\omega }_{x}\\ {-\omega }_{y} & \omega_{x} & 0 \end{array}).$$ (3)

In the following, we assume that the screw twist of the joint is already known, and we base on it the calculation of the ISA. We then illustrate the procedures to calculate: (i) the ISA; (ii) the ASA; (iii) a CS attached to the ASA; (iv) the analysis of the dispersion of the ISAs with respect to the ASA that also represents the uncertainty in the estimation of the ASA.

### Definition of the instantaneous screw axis

**Fig 1:**
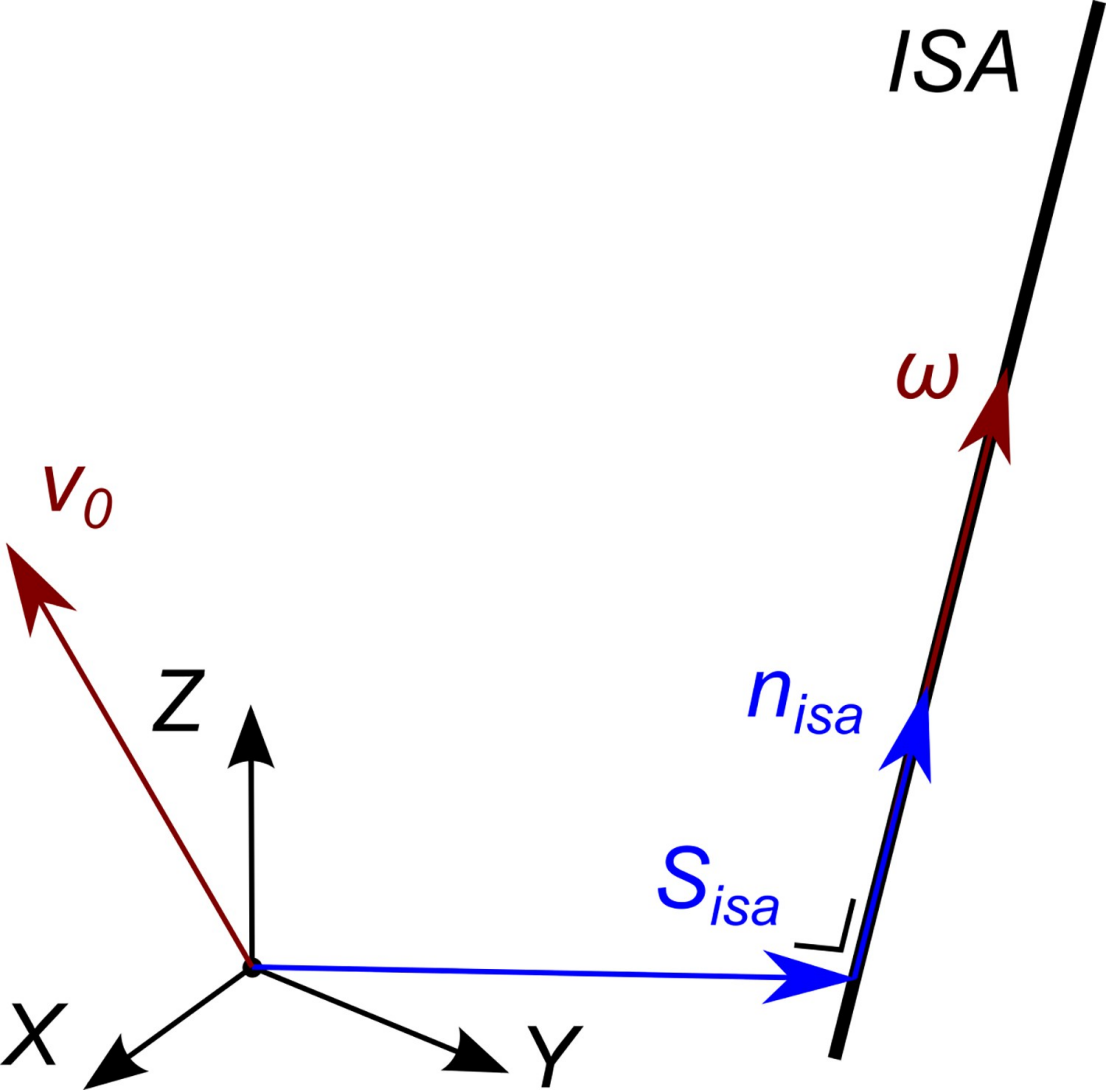
Definition of the ISA with respect to the world coordinate system.

According to the Mozzi-Chasles theorem [4, 5] every rigid-body motion can be described as a rotation and translation along an axis in space. At the velocity level, we refer to this axis as the ISA. The axis can be defined by an orientation ***n***_*isa*_ and a point ***S***_*isa*_ lying on the axis (Fig 1) that are obtained according to the following equations [7]. The orientation ***n***_*isa*_ is given by:

$$n_{isa}=\frac{\omega }{∥\omega ∥},$$ (4)

while the position of the axis is defined by the point closest to the origin of the reference CS, ***S***_*isa*_, which can be found as follows:

$$S_{isa}=\frac{\omega \times v_{0}}{∥\omega {∥}^{2}}.$$ (5)

The orientation vector and the point calculated according to the previous equations are expressed within the same coordinate system in which the screw twist was expressed. When the angular velocity is low, the direction of the ISA becomes ill defined.

### Calculation of the average screw axis

Given a set of ISAs, the goal is to find the axis that is the “best fit” of the set and can be assumed as the average one. Intuitively, the orientation of the ASA, ***n***_*asa*_, is the one that is “most parallel” to the set of ISAs; the position, ***S***_*asa*_, is the pseudo-intersection point of the ISAs, i.e. the point closest to all the ISAs.

In our approach, we exploit an optimization procedure to find these quantities and to handle the samples where the angular velocity is low.

**Fig 2:**
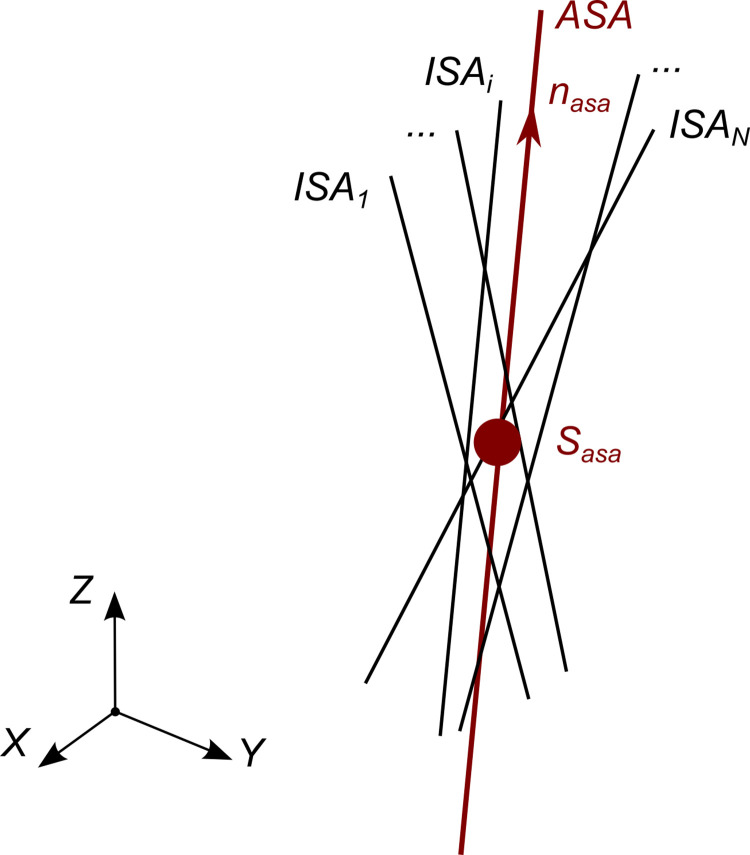
Definition of the ASA calculated as the average orientation of the ISAs and the optimal pseudo-intersection of the ISAs.

The orientation (Fig 2) of the ASA can be obtained as follows: for a given set of *N* rotational velocity vectors ***ω***_*i*_ with *i* = 1,2,…,*N*, we look for the unit vector ***n***_*asa*_ that is the most parallel to all rotational velocities:

$$\begin{array}{c} n_{asa}=\underset{n}{\mathrm{argmax}}f(n)=\underset{n}{\mathrm{argmax}}\left(\frac{1}{N}\sum_{i=1}^{N}{\left(\omega_{i}\cdot n\right)}^{2}\right)\\ \mathrm{subject}\;\mathrm{to}\;\|n\cdot n\|=1 \end{array}$$ (6)

The objective function *f*(***n***) considers the squared value of the scalar product between each rotational velocity **ω**_*i*_ and the unknown orientation ***n***_*asa*_. The constraint ensures that ***n***_*asa*_ is a unit vector. This optimization problem can be rewritten into the following form:

$$n_{asa}=\underset{n}{\mathrm{argmax}}\frac{n^{T}C_{\omega }n}{n^{T}n},$$ (7)

Where *C*_**ω**_ represents the covariance matrix associated with the angular velocity:

$$C_{\omega }=\frac{1}{N}\sum_{i=1}^{N}\left(\omega_{i}\omega_{i}^{T}\right).$$ (8)

This form of optimization problem can be shown to correspond to an eigenvalue maximization problem [28]. The global maximizer ***n***_*asa*_ is the eigenvector of *C*_**ω**_ that corresponds to the largest eigenvalue. Remark that the eigenvalues are always real since *C*_**ω**_ is a real symmetric matrix.

The position ***S***_*asa*_ (Fig 2) can be calculated via an optimization procedure similar to the one adopted for ***n***_*asa*_.

The average intersection point, ***S***_*asa*_, is defined for a given set of *N* rotational velocity vectors **ω**_*i*_ and *N* translational velocity vectors **v**_0,*i*_ with *i* = 1,2,…,*N*, as the point with the smallest average squared velocity due to all the twists (**ω**_*i*_, **v**_0,*i*_):

$$S_{asa}=\underset{S}{\mathrm{argmin}}\left(\frac{1}{N}\sum_{i=1}^{N}{∥\omega_{i}\times S+v_{0,i}\|}^{2}\right).$$ (9)

Problem (9) is a linear least squares regression problem in the variable ***S***. A general linear regression model has the following form *Y* = *Xβ*+*η*, where *Y* is the vector of observations, *X* the matrix of predictors, *β* the parameters to be estimated and *η* the vector of residuals [29]. *β* is estimated by minimizing the sum of the squared residuals:

$$\hat{\beta }=\underset{\beta }{\mathrm{argmin}}{\;(Y\;-X\beta )}^{T}(Y\;-X\beta ),$$ (10)

which has the following closed-form solution:

$$\hat{\beta }={{(X}^{T}X)}^{-1}X^{T}Y.$$ (11)

In our case, the variables in the regression model correspond to:

$$\begin{array}{c} Y=\frac{1}{\sqrt{N}}\left(\begin{array}{c} v_{0,1}\\ v_{0,2}\\ \ldots \\ v_{0,N} \end{array}\right);\;X=\frac{1}{\sqrt{N}}\left(\begin{array}{c} {-[\omega_{0}]}_{\times }\\ -{\left[\omega_{1}\right]}_{\times }\\ \ldots \\ -{\left[\omega_{N}\right]}_{\times } \end{array}\right);\;\beta =S;\\ \eta =\frac{1}{\sqrt{N}}(\begin{array}{c} {v_{0,1}+[\omega_{0}]}_{\times }S\\ v_{0,2}+{\left[\omega_{1}\right]}_{\times }S\\ \ldots \\ v_{0,N}+{\left[\omega_{N}\right]}_{\times }S \end{array}). \end{array}$$ (12)

Filling in these variables in the closed-form solution (11) leads to the following solution for the average intersection point ***S***_*asa*_:

$$S_{asa}={\left(\sum_{i=1}^{N}{\left[\omega_{i}\right]}_{\times }{\left[\omega_{i}\right]}_{\times }^{T}\right)}^{-1}\sum_{i=1}^{N}\left({\left[\omega_{i}\right]}_{\times }v_{0,i}\right),$$ (13)

where we made use of the property ${\left[\omega_{i}\right]}_{\times }^{T}={-[\omega_{i}]}_{\times }$ in the right-hand side of the equation.

The matrix inverse in (13) is ill-defined when the matrix is rank-deficient. This happens when all rotational velocities have the same orientation (ISA remains parallel to itself) or when the rotational velocities are zero, resulting in many possible solutions corresponding to a minimum. This problem can be resolved by adding the following regularization term to the objective function in (9):

$$\epsilon ∥(S-S_{0}){∥}^{2},$$ (14)

which biases the optimization problem towards a given prior value **S**_0_ in the directions which are ill-defined. The prior value should be chosen as an approximation of the geometrical joint center provided this information is available. Alternatively, the barycenter of the marker cluster or the origin of the coordinate system attached to one segment of the joint can be entered as prior value. This regularization has only a minor influence on the result in case of well-defined problems.

The *ϵ* is a weighting parameter for the contribution of the regularization term to the main cost function (9) of the optimization problem. The resulting problem corresponds to a damped or regularized least squares problem. Using a similar derivation as (11), the solution of the regularized optimization problem is found as:

$$S_{asa}={\left([\frac{1}{N}\sum_{i=1}^{N}{\left[\omega_{i}\right]}_{\times }{\left[\omega_{i}\right]}_{\times }^{T}]+\epsilon I_{3}\right)}^{-1}(\frac{1}{N}\sum_{i=1}^{N}\left({\left[\omega_{i}\right]}_{\times }v_{0,i}\right)+\epsilon S_{0}).$$ (15)

## Confidence analysis of the ASA with respect to the ISAs

The uncertainty of the ASA calculation can be studied in terms of the variance observed in ISA orientation and position. It can be interpreted as a measurement of the goodness of fit of the ASA with respect to the ISAs.

### ISA orientation

**Fig 3:**
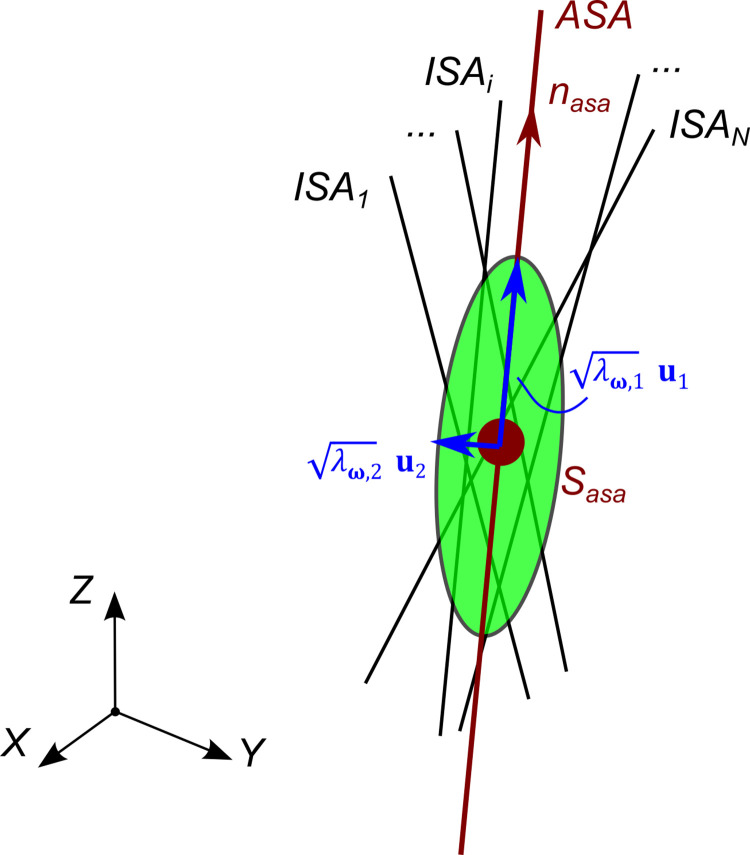
Analysis of the dispersion of the ISAs with respect to the ASA, represented as the confidence ellipsoid.

The dispersion of the ISA orientation (Fig 3) with respect to the *n*_*ASA*_ can be analyzed through the covariance matrix *C*_**ω**_ of **ω**_*i*_ (8). Since *C*_**ω**_ is a real symmetric matrix, its eigenvalue decomposition can be written as follows:

$$C_{\omega }=U_{\omega }*\Lambda_{\omega }*U_{\omega }^{T},$$ (16)

where $U_{\omega }=[\begin{array}{ccc} u_{1} & u_{2} & u_{3} \end{array}]$ is a 3×3 orthogonal matrix containing the eigenvectors and *Λ*_**ω**_ is a real diagonal matrix containing the eigenvalues *λ*_**ω**,1_, *λ*_**ω**,2_, and *λ*_**ω**,3_ in decreasing order of magnitude with units $\left[\frac{rad^{2}}{s^{2}}\right]$. The first eigenvector **u**_1_ corresponds to the average orientation of the ISA. The first and second eigenvectors **u**_1_ and **u**_2_ in *U*_**ω**_ identify the plane where most of the ISA variations occur. Consequently, the third eigenvector **u**_3_ indicates the normal vector to this plane. Based on the eigenvalues and eigenvectors, it is possible to calculate the confidence ellipsoid, i.e., the ellipsoid that contains the rotational velocity vectors **ω**_*i*_ within a confidence level α. The direction and magnitude of the ellipsoid axes give information about the dispersion of the ISAs with respect to the ASA. The directions of the axes of the ellipsoid are given by the eigenvectors *U*_**ω**_. Considering the eigenvalues:

$$\Lambda_{\omega }=\left[\begin{array}{ccc} \lambda_{\omega 1} & 0 & 0\\ 0 & \lambda_{\omega 2} & 0\\ 0 & 0 & \lambda_{\omega 3} \end{array}\right]$$ (17)

and the associated standard deviation:

$$\sigma_{\omega i}=\sqrt{\lambda_{\omega i}}$$ (18)

the magnitude of the i-th axis of the ellipsoid is then given by:

$$m_{\omega i}=\sqrt{\gamma_{\alpha ,3}}*\sigma_{\omega i}$$ (19)

where *γ*_*α*,3_ is the value of the inverse *χ*^2^ probability distribution with significance α = 0.95 and 3 degrees of freedom.

When the ratio of the sum of the second and third eigenvalues over the first decreases and approaches zero, it can be concluded that the ISA tends to one dominant direction **u**_1_. When the ratio of the third eigenvalue over the second decreases, the ISAs tend to lie in a dominant plane spanned by **u**_1_ and **u**_2_. The two ratios can be defined as follows:

$$\begin{array}{c} \rho_{1}=\sqrt{\frac{\lambda_{\omega ,2}+\lambda_{\omega ,3}}{\lambda_{\omega ,1}}}\\ \rho_{2}=\sqrt{\frac{\lambda_{\omega ,3}}{\lambda_{\omega ,2}}} \end{array}$$ (20)

### ISA position

For a linear regression model of the form *Y* = *Xβ*+*η*, where the variance *var*(*η*) is assumed to be isotropic and equal to *σ*^2^*I*, the variance on the estimated *p*×1 parameter vector $\hat{\beta }$ can be determined as follows [29]:

$$var(\hat{\beta })=\sigma^{2}{{(X}^{T}X)}^{-1}.$$ (21)

The value of *σ*^2^ is unknown in practice, but can be estimated from the *n*×1 vector of residuals

$\hat{\eta }=(Y-X\hat{\beta })$ as follows:

$${\hat{\sigma }}^{2}=\frac{{\hat{\eta }}^{T}\hat{\eta }}{n-p}.$$ (22)

Applying these general equations to the least squares problem of finding the ASA position, the variance on the position, referred to as *C*_**S**_, is worked out as follows for the unregularized case:

$$C_{S}=var(S_{asa})=\sigma^{2}{\left[\sum_{i=1}^{N}\frac{1}{N}{\left[\omega_{i}\right]}_{\times }{\left[\omega_{i}\right]}_{\times }^{T}\right]}^{-1}.$$ (23)

Assuming that the variance on the translational velocity residual *η* is isotropic and equal to *σ*^2^*I*, the value of *σ*^2^ can be estimated by substituting the velocity residuals as defined in (9) into (22) for ***S*** = ***S***_*asa*_ and with *n* = 3*N* and *p* = 3:

$${\hat{\sigma }}^{2}=\frac{\sum_{i=1}^{N}{\left(\omega_{i}\times S_{asa}+v_{0,i}\right)}^{T}(\omega_{i}\times S_{asa}+v_{0,i})}{N(3N-3)}.$$ (24)

The value of *σ*^2^ is influenced by two types of uncertainty. One type of uncertainty is due to measurement noise on the recorded twists. The other type of uncertainty is caused by changes in the position of the ISAs throughout the batch. The covariance matrix *C*_**S**_ would ideally only capture the latter source of uncertainty. Unfortunately, there is no way to distinguish between the two sources of uncertainties solely from the velocity residuals. Despite this drawback, the covariance matrix *C*_**S**_ is still useful for studying the main directions of uncertainty for the ASA position. This is done by using the eigenvalue decomposition on the covariance matrix *C*_**S**_ as previously described for the orientation:

$$C_{S}=U_{S}*\Lambda_{S}*U_{S}^{T},$$ (25)

with orthogonal matrix *U*_**S**_ as the eigenvectors and the eigenvalues in *Λ*_**S**_ in decreasing order of magnitude. The first eigenvector in *U*_**S**_ will then indicate the direction with the highest uncertainty on the ASA position. The procedure for determining the confidence ellipsoid is the same as for the orientation. Remark that the corresponding ratios of the eigenvalues *ρ*_1_ and *ρ*_2_ are independent of the estimation of *σ*^2^.

By inspecting the formula for the covariance matrix of the position *C*_**S**_ (25) and the covariance matrix of the orientation *C*_**ω**_ (8), the following relationship between the two can be derived:

$$C_{S}=\sigma^{2}{\left(C_{\omega }-trace(C_{\omega })\right)}^{-1}.$$ (26)

Since *C*_**S**_ and *C*_**ω**_ are both symmetric matrices, they share the same eigenvectors. Consequently, the direction with the highest uncertainty on the ASA position corresponds to the average direction of the rotational velocities, *i*.*e*., the orientation of the ASA.

### Coordinate system attached to the ASA

Once the ASA is determined, it is possible to define a local coordinate system based on it. Such a coordinate system will be entirely determined in a functional way and exploits the eigenvalues decomposition of the covariance matrix associated to the angular velocity:

■ Origin ***O*** ≔ ***S***_*asa*_
■ ${\hat{e}}_{1}$: ***n***_*asa*_ i.e. the eigenvector belonging to the largest eigenvalue.
■ ${\hat{e}}_{2}:$ the 3^rd^ eigenvalue of the covariance matrix of the **ω** vector (8).
■ ${\hat{e}}_{3}:{\hat{e}}_{1}\times {\hat{e}}_{2}$ to ensure a right-handed frame.

The direction ${\hat{e}}_{2}$ represents the normal to the plane containing the largest variation of the ISA. The ASA CS is then represented in the form of a 4x4 homogeneous matrix:

$${}^{ASA}T=[\begin{array}{cccc} \vdots & \vdots & \vdots & \vdots \\ {\hat{e}}_{1} & {\hat{e}}_{2} & {\hat{e}}_{3} & O\\ \vdots & \vdots & \vdots & \vdots \\ 0 & 0 & 0 & 1 \end{array}]$$ (27)

## Summary of the method

The different steps in the methodology for calculating the average screw axis and quantifying its uncertainty are summarized to enhance the replicability of the method. Starting from a batch of relative poses describing the motion of a joint, the following steps need to be followed:

1. calculate the screw twist according to Eqs (1), (2) and (3);
2. determine the average direction of the screw axis ***n***_*asa*_ as the eigenvector belonging to the largest eigenvalue of the covariance matrix as in (8);
3. determine the average intersection point of the screw axes ***S***_*asa*_ from (15) with a chosen regularization weight *ϵ* and prior value **S**_0_;
4. study the dispersion of the orientation of the ISAs by first calculating the eigenvalues of the covariance matrix in (8) according to (16) and (17), and then by considering the ratios of the eigenvalues defined in (20) or the dimensions of the uncertainty ellipsoid found from (18) and (19);
5. study the dispersion of the position of the ISAs by first calculating the eigenvalues of the covariance matrix in (23) making use of the estimate of *σ* in (24), and then, similarly as for orientation, by considering the ratios of the eigenvalues or the dimensions of the uncertainty ellipsoid.

## Calculation examples

In this section, we demonstrate the proposed approach by applying the previously described procedures to some datasets. The aims of these tests were to: (i) demonstrate the calculation of the ASA and the analysis of ISA dispersion on different datasets; (ii) study the sensitivity to noise of the new method and compare it to the current state of the art (SoA); (iii) demonstrate the effect of the regularization term.

Three datasets were tested: (i) the motion of a human knee during a gait cycle of a gait analysis exam; (ii) the motion of an artificial hinge recorded via motion capture and (iii) a numerically generated dataset representing a swing motion about a perfectly cylindrical joint.

### Description of the datasets

#### Human knee

**Fig 4:**
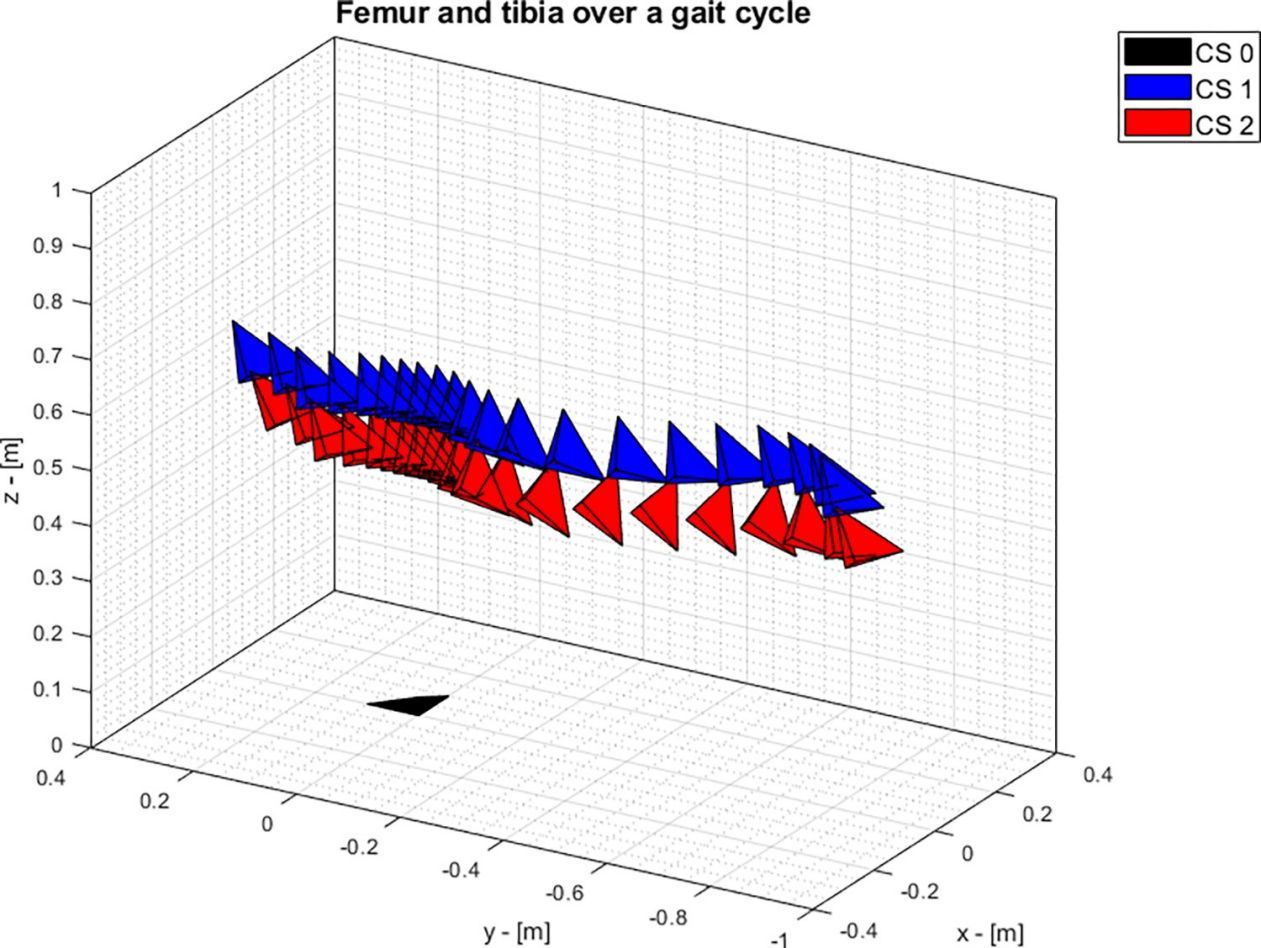
Poses of femur and tibia across one gait cycle. The motion progresses from the left to the right. CS0 is the world coordinate system, CS1 is the coordinate system attached to the femur, CS2 is the coordinate system attached to the tibia. For CS 1 and CS 2, the x-axis is pointing forward, the y-axis is pointing upwards while the z-axis is on the medio-lateral direction.

The studied data consist in the poses of the femur and the tibia of a subject that underwent a total knee arthroplasty. The implant was on the left knee. The motion was recorded over an entire gait cycle during a ground gait by means of fluoroscopy imaging. The estimated accuracy was <1 degree for all rotations, <1 mm for in-plane and <3 mm for out-of- plane translations. The original sampling frequency was 25 Hz. The analysis was limited to an entire gait cycle defined by the samples between two consecutive heel strikes. The data were part of the CAMS-Knee dataset [30] and they were visually represented in Fig 4:

#### Hinge joint

**Fig 5:**
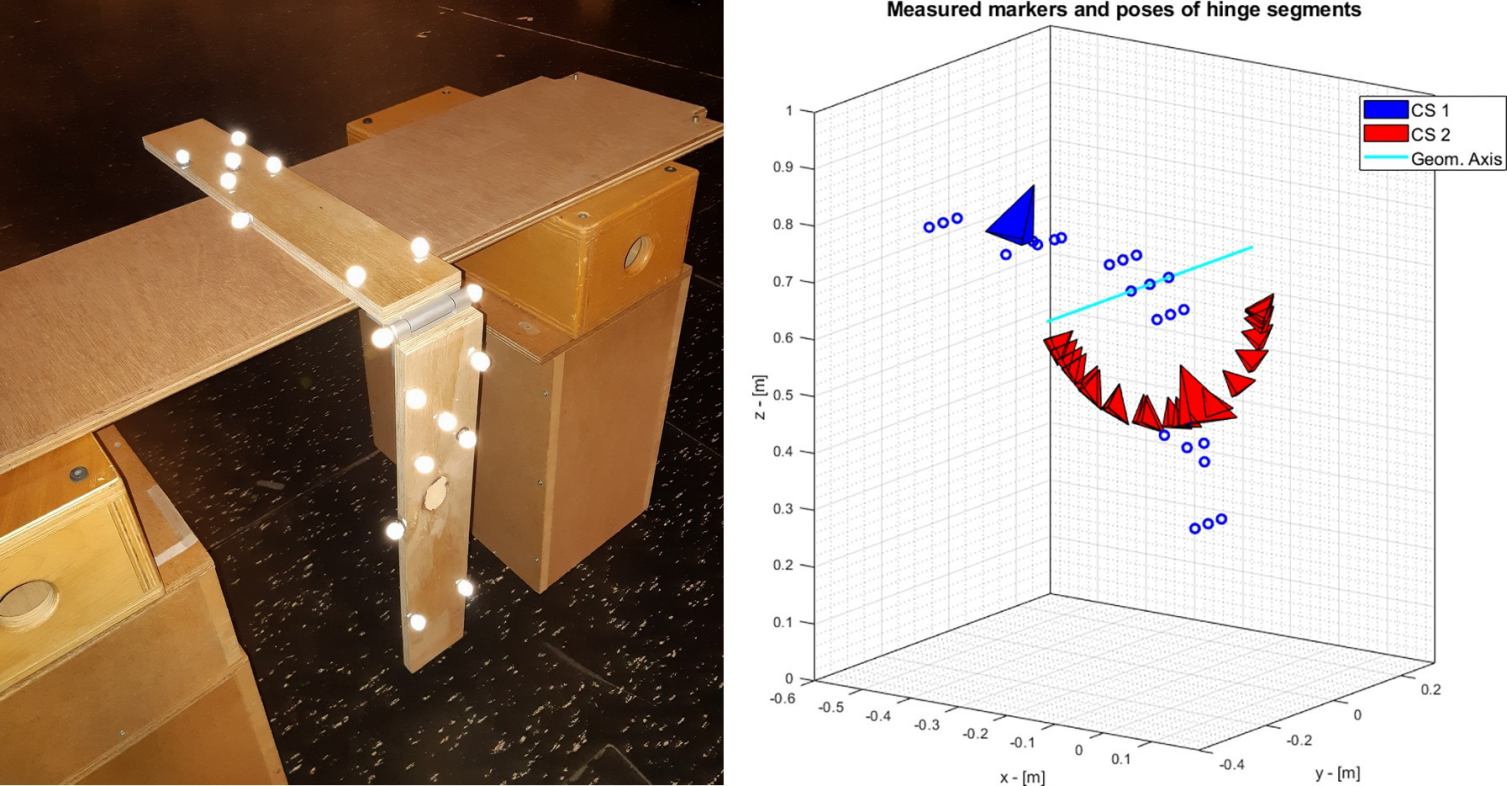
a) Experimental setup for the hinge measurements and b) the three-dimensional reconstruction of the motion of the artificial hinge. The poses of the two segments are visualized.

An artificial, custom-made hinge was left free to swing after an initial perturbation. The swing motion was recorded by means of an optoelectronic system (Vicon MX®, Oxford, UK, 10 cameras, sampling frequency 100 Hz) and passive markers attached to the hinge segments. The coordinate systems attached to each segment were reconstructed by means of the point-cluster technique [31] based on the marker measurements (Fig 5). The first swing cycle was discarded to remove possible artifacts and the analysis was limited to the second complete swing cycle.

#### Ideal cylindrical joint

**Fig 6:**
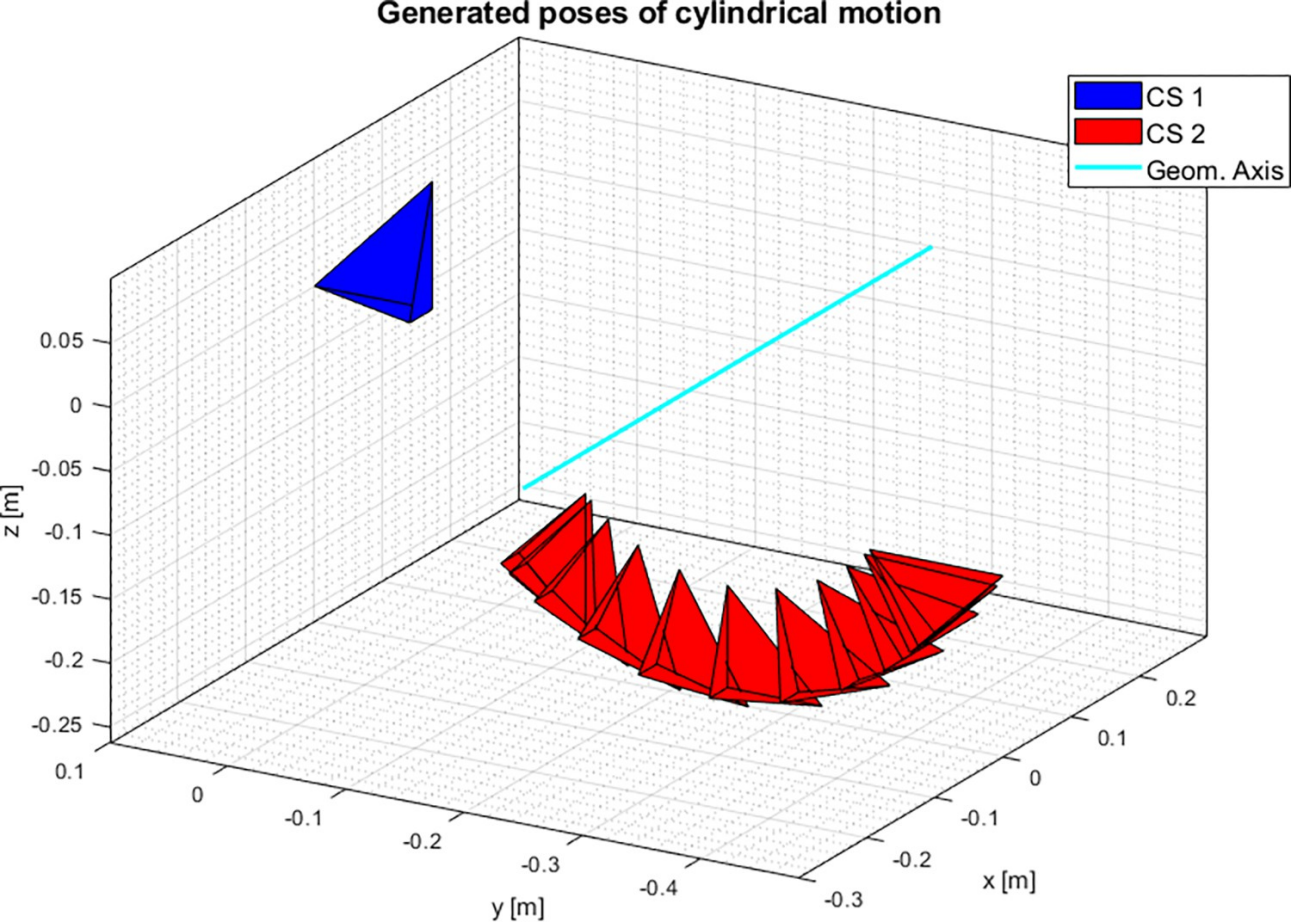
Generated data for a swing cycle about an ideal cylindrical joint.

The dataset (Fig 6) was generated as a rotational trajectory about a geometric axis (GA) defined by the following direction ***n***_***GA***_ and position ***S***_***GA***_:

$${}_{1}n_{GA}=[0\;0\;1]$$

$${}_{1}S_{GA}=[0\;-0.25\;0]m$$

The origin of CS 2 with respect to CS 1 was defined as:

$$\begin{array}{c} {}_{1}x_{c}=r*cos\;(\alpha )\\ {}_{1}y_{c}=r*sin\;(\alpha )\\ {}_{1}z_{c}=0\\ {}_{1}^{2}O_{c}={}_{1}S_{GA}+[{}_{1}x_{c}\;{}_{1}y_{c}\;{}_{1}z_{c}] \end{array}$$ (28)

where *r = 0*.*25 m* and

$$\alpha =A*\sin\left(\frac{2\pi }{T}t\right)+U$$ (29)

with: A = 45 deg.; U = 180 deg.; T = 1 s; *t*∈[0, *T*_*max*_]

The orientation of CS 2 with respect to CS 1 was defined as follows:

$$\begin{array}{c} {}_{1}^{2}j_{c}=-\frac{{}_{1}^{2}O_{c}}{\left\|{}_{1}^{2}O_{c}\right\|}\\ {}_{1}^{2}k_{c}=[0\;0\;1]\\ {}_{1}^{2}i_{c}={}_{1}^{2}j_{c}\times {}_{1}^{2}k_{c} \end{array}$$ (30)

Hence, the complete pose of CS2 with respect to CS1 is given by

$${}_{1}^{2}T_{c}=\left[\begin{array}{cccc} \vdots & \vdots & \vdots & \vdots \\ {}_{1}^{2}i_{c} & {}_{1}^{2}j_{c} & {}_{1}^{2}k_{c} & {}_{1}^{2}O_{c}\\ \vdots & \vdots & \vdots & \vdots \\ 0 & 0 & 0 & 1 \end{array}\right]$$ (31)

## Results

### Effect of the regularization

To test the effect of the regularization, we first calculated the ASA for each dataset with *ϵ* = 0 (no regularization). Then the calculation was repeated for different choices of *ϵ*. Each time, the ASA calculated with regularization was compared to the non-regularized one by means of the following parameters:

- common normal distance between the regularized and non-regularized ASAs
- distance between the position points (***S***_*ASA*_) of the regularized and non-regularized ASAs.

We did not test the effect on the direction of the ASA as, by definition, the regularization affects only the ASA position. Increasing the *ϵ* led to larger variations in the ASA position. This effect was more noticeable in the hinge dataset. In the case of the ideal cylindrical joint the regularization was very effective in picking an ASA position close to the reference one, thus a large ***S***_*ASA*_ was observed. The choice of *ϵ* did not affect the result.

The results are reported in Table 1:

**Table 1:** Effect of the regularization: Common normal distance and position point (*S*_*ASA*_) of the regularized ASA with respect to the non-regularized ASA.

Data	*ϵ*	Common-normal distance [mm]	*S*_*ASA*_ distance [mm]
Gait	0.0001	0.000	0.001
	0.001	0.001	0.010
	0.01	0.014	0.098
	0.1	0.139	0.868
Hinge	0.0001	0.000	0.146
	0.001	0.000	1.392
	0.01	0.001	9.392
	0.1	0.008	22.073
Ideal joint	0.0001	0.000	very large
	0.001	0.000	very large
	0.01	0.000	very large
	0.1	0.000	very large

The reference point for the regularization was chosen as: the geometric center of the knee in the case of gait data; the midpoint of the hinge joint in case of the hinge data; the defined geometric center of rotation in the case of the artificial data of the ideal joint.

### Sensitivity analysis

In the sensitivity analysis, we evaluated the effect of noise on the calculation of the ASA. The noise was added to the relative pose matrix ${}_{1}^{2}T$ in terms of a small random displacement and rotation according to the following equation:

$${}_{1}^{2}T_{noise}={}_{1}^{2}T*expm\left([\begin{array}{cccc} 0 & -\delta_{z} & \delta_{y} & d_{x}\\ \delta_{z} & 0 & -\delta_{x} & d_{y}\\ -\delta_{y} & \delta_{x} & 0 & d_{z}\\ 0 & 0 & 0 & 0 \end{array}]\right)$$ (32)

where the notation ${}_{1}^{2}T$ represents the homogeneous matrix of CS 2 expressed in CS 1

The ASA was then calculated based on the noisy data and compared to the one calculated without noise. For this purpose, we assumed *ϵ* = 0.01. We tested other choices of *ϵ* and we concluded that it did not significantly affect the sensitivity to noise analysis.

The comparison involved the following parameters:

1. angle between the two axes,
2. common-normal distance between the two axes,
3. distance between the position points (***S***_*ASA*_) of the two axes.

The calculation was repeated 100 times, regenerating the random noise each time. Finally, the standard deviation of each parameter across all the repetitions was reported.

The same analysis was conducted on the state-of-the-art method for calculating the ASA based on the instantaneous approach currently reported in the literature [11–13, 32]. The sensitivity to noise of the state-of-the-art method was then compared to our method. The sensitivity analysis was repeated on each dataset included in this study. Two noise levels were tested:

- 3 deg and 2 mm
- 6 deg and 4 mm

**Fig 7:**
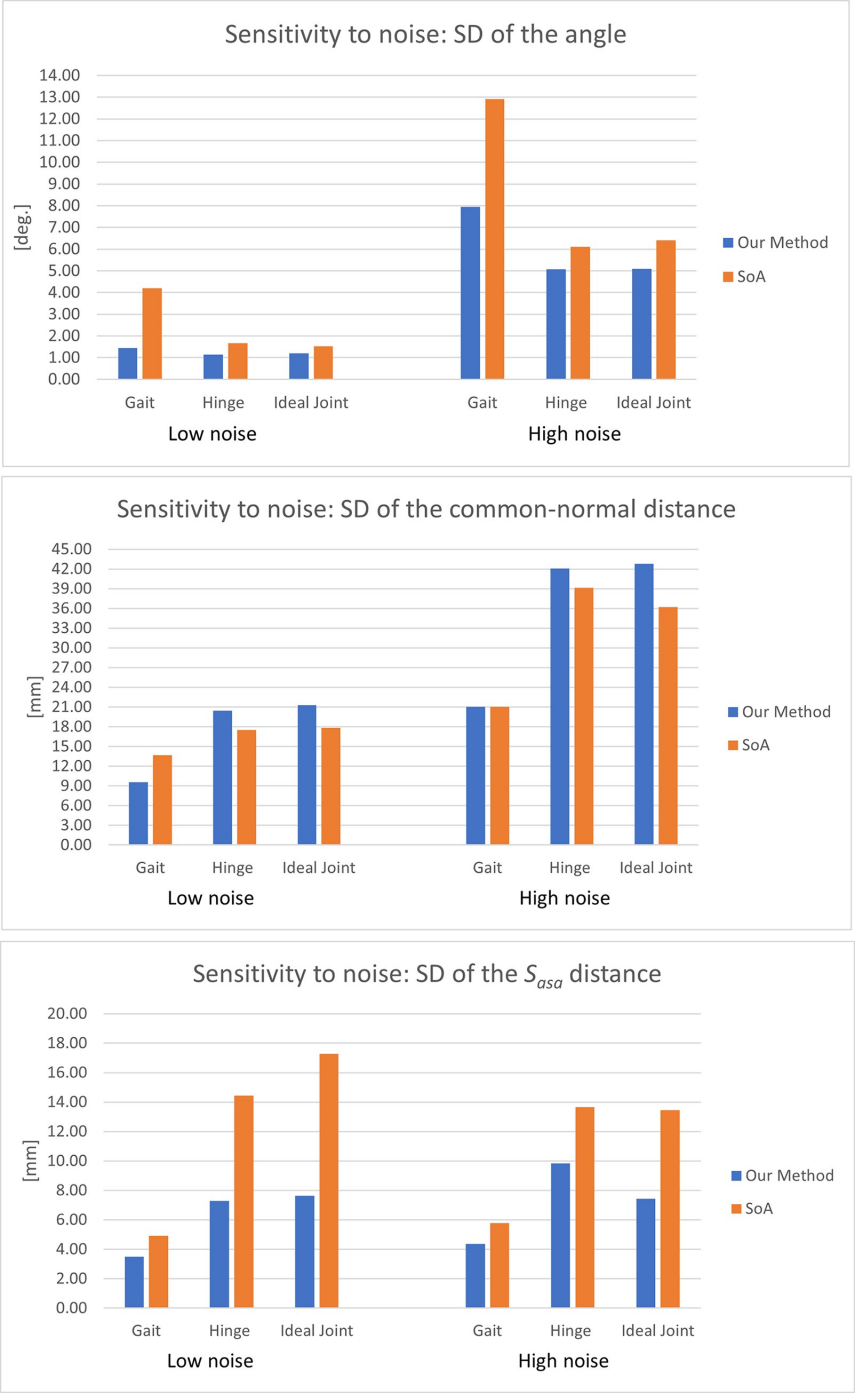
Sensitivity analysis, as calculated for ϵ = 0.01. Standard deviation (SD) of (a) the angle between the ASA with noise and without noise; (b) the common-normal distance between the two axes; (c) the ***S***_*ASA*_ distance.

The results are reported in Fig 7:

### Dispersion analysis

**Fig 8:**
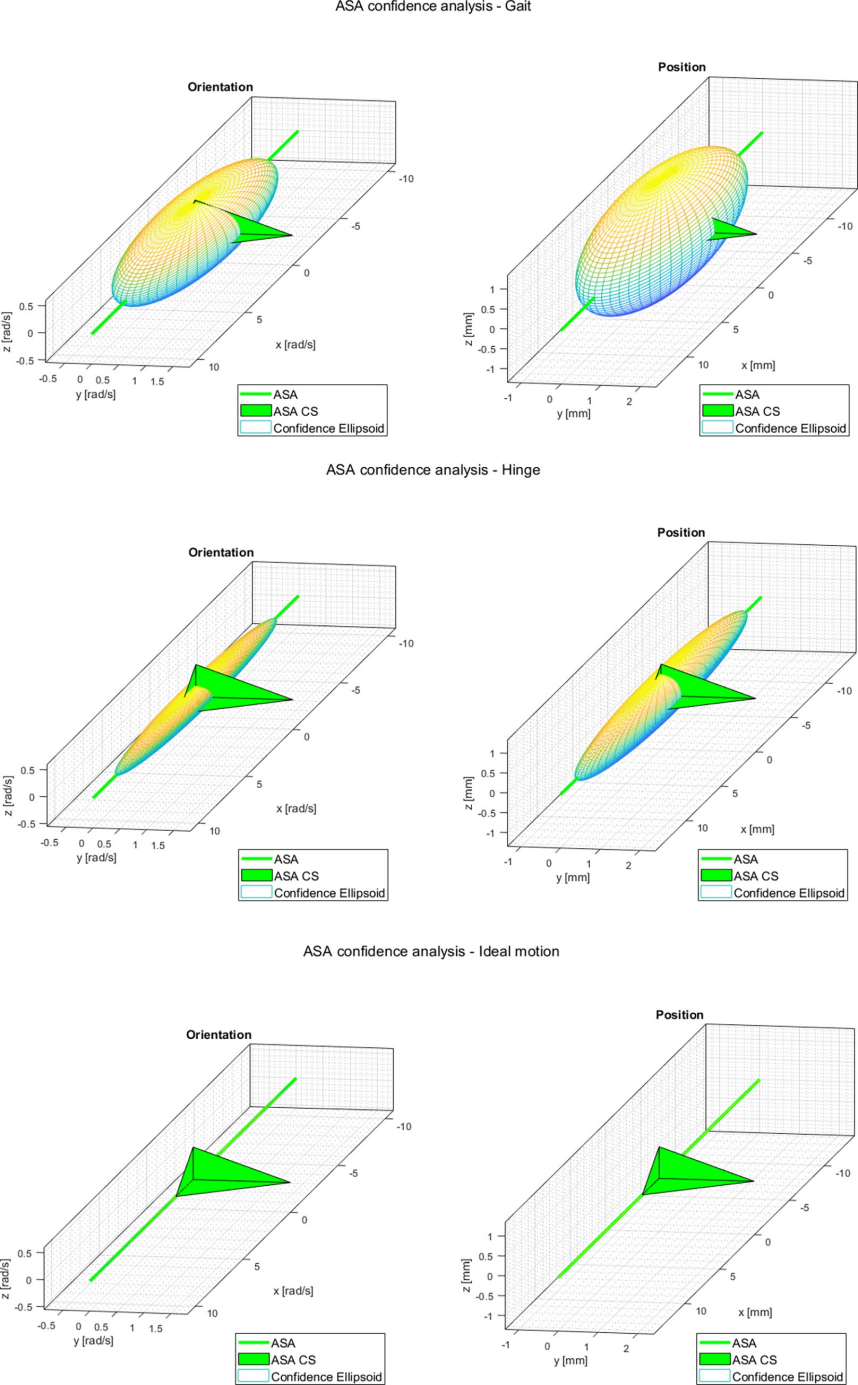
Ellipsoid of the dispersion analysis in the case of: (a) gait; (b) hinge; (c) ideal hinge, each time represented in the CS attached to the ASA. In the last case, the ellipsoid degenerated to the ASA axis itself.

The dispersion analysis was conducted on all three datasets for orientation and position of the ASA according to the methods outlined in sections 3.3.1 and 3.3.2, respectively. This analysis included the calculation of: (i) the magnitude of the axes of the confidence ellipsoids associated with the covariance matrices, *C*_**ω**_ and *C*_**s**_, of the angular velocity and ASA position; (ii) the non-dimensional ratios between the eigenvalues of these covariance matrices; (iii) the dispersion in terms of the RMSE of the orientation and of the position, according to the current literature (SoA) [12]. The results are presented in Table 2 and Fig 8.

**Table 2:** Results of the dispersion analysis.

Parameter	Gait		Hinge		Ideal cylindrical motion	
	*Direction*	*Position*	*Direction*	*Position*	*Direction*	*Position*
**Confidence ellipsoid**	**[rad/s]**	**[mm]**	**[rad/s]**	**[mm]**	**[rad/s]**	**[mm]**
Mag. 1^st^ axis	7.510	9.080	8.325	11.977	9.797	4.9e5
Mag. 2^nd^ axis	1.863	2.680	0.313	0.534	0.000	0.000
Mag. 3^rd^ axis	1.256	2.637	0.199	0.534	0.000	0.000
**Eigenvalues’ ratios**	**[]**	**[]**	**[]**	**[]**	**[]**	**[]**
*ρ*_1_	0.299	0.586	0.045	0.089	0.000	0.000
*ρ*_2_	0.674	0.984	0.636	1.000	0.906	1.000
***SoA***	**[deg]**	**[mm]**	**[deg]**	**[mm]**	**[deg]**	**[mm]**
RMSE	30.629	22.824	2.664	25.551	0.000	3.8e4

## Discussion

In this paper we presented a novel method for determining an ASA based on the instantaneous measurements of the motion of joints. The ASA was calculated in terms of its position and direction vector. The main contributions, with respect to the previous literature, were: (i) the implementation of a weighting factor corresponding to the norm of the angular velocity in order to improve the estimation of the ASA; (ii) the introduction of a regularization term to improve the estimation of the ASA position in the cases where the ISAs tend to be parallel to each other; (iii) the definition of a local coordinate system entirely based on functional measurements; (iv) the statistical quantification of the dispersion of the ISAs with respect to the ASA in terms of its variance.

A known issue in calculating the ISA and then the ASA is that the ISA is ill-defined for low angular velocities. For this reason, the previous literature recommended thresholding the angular velocity or angular displacements and to calculate the ISA and the ASA based only on the samples above the threshold [11, 12]. The choice of a threshold may significantly affect the results [33]. The method presented in this paper is meant to serve as an improvement to the methods currently proposed in the literature when the rotation axis and the center of rotation of a joint have to be estimated from functional measurements. Examples are the analysis of human joints, such as the knee or the hip; or robotics applications, such as the analysis or control of the rotation of joints. In our approach we aimed to eliminate the need for a threshold and introduced instead a weighting using the norm of the angular velocity. This was motivated by the fact that the influence on the direction of the angular velocity caused by additive isotropic noise is inversely proportional to the norm of this angular velocity. This approach leads to a more accurate estimation of the ASA that is not affected by the arbitrary choice of a threshold.

Furthermore, weighting with the norm of the angular velocity can be shown to be equivalent with calculating the average screw axis using measurements which are equidistant in the progress of the motion rather than measurements which are equidistant in time. The progress of motion should then be defined as the integral of the absolute value of the angular velocity about the ISAs, which is appropriate for hinge-like joints. Hence, such weighting makes the calculated average screw axis independent of the magnitude of the angular velocity at every time instant during the motion.

The introduction of the regularization term is aimed to improve the calculation of the ASA position in the limit cases where the ISAs tend to be parallel to each other or the cases where the position of the ASA is ill-defined. In such cases, regularization helps to keep the position of the ASA (***S***_***ASA***_) in the neighborhood of a reasonable point. Where possible, this point should be assumed as the geometrical center of the joint. However, the knowledge of this point represents only an improvement for the estimation of the ***S***_***ASA***_, but it is not strictly necessary for its calculation. The weight of the regularization term, *ϵ* in Eq (15), is an arbitrary parameter that should be kept small. Ideally, it should be experimentally tuned for each application it is intended for.

The effects of regularization were tested on three datasets. The regularization did not affect the calculation of the ASA direction, while it affected only the position. This was in accordance with the mathematical definition of the regularization term. From the experimental data, we observed that the regularization was more effective for larger *ϵ*, producing an ASA more distant from the non-regularized one, and closer to the reference point. The effect of regularization was more obvious in the case of the hinge dataset that represented a real joint with physical characteristics close to the limit case of purely rotational cylindrical motion. In this case, the regularization did not affect significantly the common-normal distance, while it had a strong impact on the position of the ASA. To further investigate the effect of regularization, we considered a simulated ideal cylindrical joint (artificial data of a purely rotational motion) where the position of the ASA is known to be ill-defined. In this case, the regularization was very effective in providing a valid S_ASA_ close to the geometric center. In this limit case, the choice of the *ϵ* had no effect on the overall result. The very large distance observed for the ***S***_***ASA***_ and the almost null common-normal distance suggested that in the non-regularized case, the ASA position was undefined along the direction of the rotation axis.

The regularization improves the estimation of the center of rotation in the cases of hinge-like joints. The regularization needs to be tuned in terms of the expected position of the center of rotation, and the weight of the regularization. The expected position of the center of rotation can be determined from geometric information, where available. For example, the knee center can be determined as the midpoint between epicondyles. When no geometric information is available, the barycenter of the marker cluster or the origin of the coordinate system attached to the marker cluster can be used.

In the sensitivity analysis, we compared our method to the SoA. With reference to Fig 7A, we observed that our approach was less sensitive to noise for estimating the direction of the ASA in the case of gait. Improved sensitivity to noise was also observed for the estimation of the ***S***_***ASA***_ in the cases of the hinge and of the ideal rotational joint (Fig 7C). For the common-normal distance (Fig 7B), our method performed worse than SoA for the hinge and ideal joint but slightly better than SoA for the gait.

Previous literature suggested to quantify the uncertainty in the calculation of the ASA, i.e. the dispersion of the ISA with respect to the ASA, by means of the RMSE of the angle and distance [12]. In our approach, we further improved such a measurement by calculating the confidence ellipsoid that provided additional information about the magnitude and the direction of the dispersion. Such dispersion was quantified both for the orientation and the position of the ISAs, with respect to the ASA. The ratios of the eigenvalues helped in quantifying the rate of deviation of the motion of a hinge-like joint from a perfect hinge case. With reference to Table 2, as expected we observed higher RMSE dispersion in ISA orientation in the case of gait, due to the expected physiological variations in the knee ISA across the gait cycle. A coherent result was observed in the confidence ellipsoid and in the ratios: for a perfect hinge we would expect *ρ*_1_ = 0, that was the case for the ideal cylindrical motion, while this value was larger for gait, meaning that the human knee behaviour deviated from a perfect hinge case. In all cases, the confidence ellipsoid suggested that the motion occurred about a dominant direction. The variation on the secondary axes suggested a uniform dispersion of the ISAs with respect to the rotation axis. The dispersion was larger in the case of gait, reduced in the case of the hinge and null in the case of the ideal cylindrical motion, where the ellipsoid degenerated to the rotation axis itself (Fig 8). In fact, in the case of ideal motion, the motion was entirely along the first axis of the ellipsoid. The null first ratio correctly detected the motion occurring only along the main axis. In such a case, the second ratio is not significant. In general, the lower the ratios, the more accurate is the estimation of the ASA while the ISAs have a smaller dispersion with respect to the ASA. The largest variation in ISA position was along the rotation axis, i.e. the 1^st^ axis of the ellipsoid, for all the cases considered. The positional error in the case of the ideal motion was very high due to the fact that the instantaneous position of the ISA was ill-defined, as expected for a purely rotational motion. As expected, the variations in the position of the ISA were larger in the case of gait compared to the hinge.

The main limitation of this study is in the limited cases that were tested. For a better application and interpretation of the results in a biomechanical context, further cases should be investigated, with special reference to other anatomical joints such as the ankle or the elbow. In this context, the methods here proposed may be exploited to characterize healthy and pathological joints. The performance of this new method should be evaluated in each case.

## Conclusion

In this paper, we presented a novel method for calculating: (i) an average screw axis, (ii) the position of the center of rotation of a joint and (iii) a method to quantify the accuracy of the ASA and the dispersion of the ISAs with respect to the ASA. The introduction of a regularization factor allowed to provide working results even in the limit cases where the ASA is ill-defined, such as purely rotational motion. The tests showed that our method was able to successfully estimate the rotation axes and had an improved robustness to additive noise when compared to the current SoA method, especially in the limit-case of the cylindrical joint. The method we proposed is recommended for estimating the ASA of hinge-like joints, such as the human knee especially in presence of noisy data.
